# Production and evaluation of the chemical and mechanical properties of nanocellulose and nanowood starch‐based biodegradable films potential candidates for moisture absorbers for food packaging

**DOI:** 10.1002/fsn3.2194

**Published:** 2021-02-21

**Authors:** Zahra Ebadi, Hamidreza Ghaisari, Behjat Tajeddin, Seyed Shahram Shekarforoush

**Affiliations:** ^1^ Department of Food Hygiene and Public Health School of Veterinary Medicine Shiraz University Shiraz Iran; ^2^ Agricultural Research, Education and Extension Organization (AREEO) Animal Science Research Institute (ASRI) Karaj Iran; ^3^ Agricultural Research Education and Extension Organization (AREEO) Agricultural Engineering Research Institute (AERI) Karaj Iran

**Keywords:** biodegradable films, moisture‐absorbent pad, nanocellulose, nanowood

## Abstract

This study was conducted to prepare starch‐based moisture absorbent pads from nanocellulose (NC) and nanowood (NW) particles using solution casting evaporation method and to evaluate their physical and mechanical properties at different thicknesses. The swelling degree (*SD*), water vapor permeability (WVP), tensile strength (TS), and elongation at break (EB), of prepared biofilms were measured. Structural properties of biofilms were evaluated by X‐ray diffraction (XRD) and scanning electron microscopy (*SEM*). Results indicated that two types of biopolymers showed the highest level of *SD* at thicknesses lower than 100 µm. The highest level of *SD* in the lowest time belonged to nanowood biofilm. Nanowood biofilms also showed highest WVP at lower thicknesses. Due to the highest EB and the lowest TS values, improvement was observed in mechanical properties of both nano biofilms. The high hydration capacity and WVP of low‐thickness NW films make it a promising candidate for developing biodegradable films with the potential to be used as a moisture‐absorbing pad in active food packaging.

## Graphical abstract

This study was conducted to prepare starch‐based moisture absorbent pads from nano cellulose and nanowood particles using solution casting evaporation method. Nano wood film is suitable candidate for the production of degradable films with the potential to be used as a moisture absorbing pad in active food packaging.

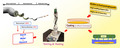

## 1 INTRODUCTION

Cellulose as the most widespread polymer in plants is the most plentiful organic material on the earth (Payne et al., 2015). Due to the fibers’ properties such as eco‐friendly, low cost, and biodegradable nature, it is suitable for development of biofilms as food packaging materials (De Azeredo, 2009). However, the use of biopolymers is limited because of weak and poor mechanical and barrier characteristics. Therefore, one of the research priorities of scientists is to improve four basic functions of biopolymer food packages including protection and preservation, containment, convenience, and marketing (Petersen et al., 1999; Radusin et al., 2016; Sorrentino et al., 2007).

Considering the effect of the moisture content and water activity on food deterioration (Vermeiren et al., 1999), one of the key properties of food packages is their water activity and water vapor permeability (WVP). Under some conditions such as temperature fluctuation, drip loss, and biochemical reactions (breakdown of fats and carbohydrates) (Labuza, 1996), moisture is collected in the package which will lead to the microbial growth. One possible solution for preventing this problem is to use films with proper WVP, desiccating films, or moisture‐controlling sachets or pads. Nowadays, desiccants are effectively used for a wide range of food products including meats, cheeses, and nuts (Biji et al., 2015; Ozdemir & Floros, 2004; Vermeiren et al., 1999).

Moreover, active compounds including scavengers of O_2_, CO_2_, and ethylene, moisture regulators, antimicrobial agents, antioxidants, and aroma can be packed into the sachets or pads and then directly added to the package or packaging material (Mohan et al., 2010). Commercially super absorbent polymers for liquid water control are used for high a_w_ foods. These drip‐absorbent sheets contain polymers of polyacrylate salts fixed between two plastic film layers which are highly permeable to water vapor (Mohan et al., 2010; Suppakul et al., 2003). The other common absorbent materials are silica gel, natural clay, calcium oxide, and modified starch (Labuza & Breene, 1989; Mohan et al., 2010; Rooney, 1995; Suppakul et al., 2003). The meat exudate absorbent pads were studied by Oral et al. (2009) for packaged meats and poultry which had three layers made of perforated polyethylene, cellulose, and polyethylene. Shirazi and Cameron (1992) reported that the shelf life of packaged tomato at 20°C was extended from 5 to 15–17 days with a bag having NaCl, mainly by obstruction of surface mold growth.

Over the last few years, the use of cellulose and its nanoscale particles to develop natural polymers useful for film‐forming and coatings has been widely studied. However, much less has been published on their potential to be used as biodegradable moisture absorbent pads. The present paper, therefore, aimed to develop cellulose‐based pads using nanocellulose (NC) and nanowood (NW) biofilms and to evaluate their physical and mechanical properties at different thicknesses.

## 2 MATERIALS AND METHODS

### 2.1 Materials

Two different types of fibers including NC and NW were provided by Nano Novin Polymer Co. (Sari, Mazandaran, Iran). NC fiber (gel 2.5 wt %) prepared from commercial pure cellulose fibers of softwoods and NW fiber (gel 2.5 wt %) was made from *Paulownia fortunei* wood. They were then prepared according to the methods described by Yousefi et al. (2018), Yousefi et al. (2018). The NW was composed of 50% cellulose, 30% lignin, 13% hemicellulose, and 7% extractives.

Calcium chloride anhydrous was obtained from Duksan Co., Korea. Magnesium nitrate and glycerol were purchased from Fluka Co. (Buchs, Switzerland) and Merck Co. (Darmstadt, Germany), respectively. Corn starch containing 73% amylopectin and 27% amylose was purchased from Sigma‐Aldrich (S4126, EC: 232–679–6), USA.

### 2.2 Biofilm preparation

The biofilms of NC and NW were prepared using Chaichi et al. (2017) method, with some modifications. For each g of filler (corn starch), 0.05 g nanobiofibers and 0.75 g glycerol were applied. In brief, the NC and NW fibers (0.15 g) were dispersed in distilled water (40 ml) and stirred at room temperature for 30 min at 1,000 rpm by heater‐ stirrer (Heidolf, Standard Hei, Germany). After complete dissolution, glycerol (2.25 g) was added and stirred again for 30 min. In parallel, corn starch (3 g) was dissolved in distilled water (50 ml) by stirring at room temperature. The solutions were then mixed, the volume was adjusted to 100 ml with distilled water and stirred again at 85°C at 1,000 rpm for 30 min. Then, the nanosuspensions were sonicated for 30 min at 80% amplitude and 24 kHz using ultrasound equipment (Hielscher, Model UP 200‐240H, Germany). The sonicated solutions were finally poured into petri dishes (diameter of 9 cm) and allowed to dry for 3–4 days at room temperature. The dried biofilms were removed from the plates, and placed in a desiccator containing saturated magnesium nitrate solution at 25°C and 52.8% relative humidity (RH) for at least 48 hr. The following equation was used to prepare the different thicknesses of biofilms, which was obtained from the results of multiple measurements.

(1) $$S=0.105\;B+0.20\;(R^{2}=0.997)$$

where *S* is the amounts of solution (g) and *B* is the biofilm thickness (µm)

### 2.3 Biofilm characterization

#### 2.3.1 Thickness

The thickness of biofilms was measured at 5 random positions using a digital micrometer (Mitutoyo Co., Japan), with an accuracy of 1 µm.

#### 2.3.2 Swelling degree (SD)

The 2 cm × 2 cm pieces of biofilms were weighed and immersed in distilled water. The *SD* of the samples was measured using Equation 2 (Lavorgna et al., 2010) at intervals of 2, 30, and 60 min; and 24 and 48 hr.

(2) $$SD=\frac{W_{f}‐W_{i}}{\text{Wi}}$$

where W_f_ and W_i_ are the final and initial weights (g) of the samples, respectively.

#### 2.3.3 WVP

The WVP values were determined according to the ASTM Method E96‐00 (ASTM, 2007b) as described by Chaichi et al. (2017). At first, glass permeation cups were filled with 8 g anhydrous calcium chloride desiccant to create a 0% RH storage condition and the surfaces of the cups were covered with films and sealed with molten paraffin and weighted. Then the cups were placed in a desiccator containing magnesium nitrate to create 52.8% RH at 25°C. The RH difference between two sides of the films creates a vapor pressure equal to1706.57 Pa. The cups were weighted at 2 hr intervals during at least 3 days by a digital balance (0.0001 g accuracy). The slope of the weight gain versus time was obtained by linear regression. Water vapor transition rate (WVTR) and water vapor permeability were calculated using Equations 3 and 4, respectively:

(3) $$\text{VTR =}\;\frac{\text{Curve}\;\;\text{slope}}{\text{Film}\;\;\text{area}}$$

(4) $$\text{WVP}=\frac{\text{Thickness}\times \text{WVTR}}{\text{Pressure}\;\;\text{difference}}$$

### 2.4 Mechanical properties

The biofilms were cut into 4 cm × 1 cm rectangular strips and were conditioned at 25°C and 52.8% relative humidity. Then they analyzed using a texture analyzer (Hounsfield, Model H5KS, UK) with 500 N load cell (ASTM, 2007a). The initial grip spacing and crosshead speed were, respectively, set at 20 mm and 10 mm/min. Elongation at break (EB) and tensile strength (TS) were calculated using Equations 5 and 6, respectively:

(5) $$\text{EB\textbackslash{}\% =}\;\frac{\text{Final}\;\;\text{length}‐\text{Initial}\;\;\text{length}}{\text{Initial}\;\;\text{length}}\times 100$$

(6) $$\text{TS}\;\left(\text{MPa}\right)=\frac{\text{Maximum}\;\;\text{force}}{\text{Film}\;\;\text{thickness}\times \text{Film}\;\;\;\;\text{width}}$$

### 2.5 Structural properties

#### 2.5.1 X‐ray diffraction (XRD)

The XRD patterns were observed in the angular range of 5–80° (2*θ*) using a Philips X'Pert‐MPD diffractometer (Panalytical, Netherlands) with a Cu Kα radiation (λ = 0.154 nm) operating at 40 kV and 40 mA. The interplanar spacing was calculated using Bragg's Law: 2*d* sin *θ = nλ*. Where *d* is the interplanar spacing (°A), θ is the angle of diffraction (°), *λ* is the wavelength (nm), and n is the reflection order (Koo, 2006).

#### 2.5.2 Scanning electron microscopy (SEM)

The surface and cross‐section structure of biofilms were observed following freezing under liquid nitrogen, fracturing, mounting, and coating with gold (2 min on a sputter coater) using a scanning electron microscope (VEGA\\, TESCAN, Czech Republic) operating at an accelerating voltage of 10 kV and different magnification (1,000, 5,000, and 15,000 x).

Energy Dispersive X‐ray Spectroscopy (EDXS) (INCA‐Oxford instruments‐England) was performed in conjunction with *SEM* for the elemental analysis of the samples.

### 2.6 Statistical analysis

A completely randomized design with factorial arrangement was used for statistical analysis. The analysis of variance was performed using a general linear model (GLM) within SAS package (SAS, 2013).

## 3 RESULTS AND DISCUSSION

### 3.1 SD

The *SD* of biofilms at different thicknesses and time intervals are shown in Table 1. Water uptake by biofilms was found to be reversely dependent on the thickness. As the highest SDs were obtained at thicknesses lower than 100 µm (*p* <.05). It can be at least partly attributed to the lower surface area, and in turn, lower interaction with water molecules at higher thicknesses (Cordeiro et al., 2011).

**TABLE 1:** Mean ± *SE* of swelling degree (*SD*) of nano biofilms in different thicknesses and time intervals

Biofilms	Time intervals	Thickness (µm)				
		60	90	140	180	240
Nanocellulose	2 min	3.63 ± 0.19^a^	4.21 ± 0.35^a^	3.10 ± 0.11^ab^	1.86 ± 0.56^bc^	1.13 ± 0.51^c^
	30 min	4.13 ± 0.17^a^	4.55 ± 0.06^a^	3.87 ± 0.18^a^	2.27 ± 0.53^b^	1.53 ± 0.32^b^
	60 min	4.60 ± 0.59^a^	4.80 ± 0.46^a^	3.28 ± 0.01^ab^	2.65 ± 0.35^bc^	1.68 ± 0.39^c^
	24 hr	5.57 ± 0.85^a^	4.82 ± 0.01^ab^	3.35 ± 0.27^bc^	2.31 ± 0.24^c^	1.98 ± 0.54^c^
Nanowood	2 min	5.50 ± 1.04^a^	2.61 ± 0.62^b^	2.67 ± 0.06^b^	2.09 ± 0.09^b^	1.52 ± 0.40^b^
	30 min	4.55 ± 0.66^a^	4.23 ± 0.80^a^	3.16 ± 0.04 ^ab^	2.12 ± 0.23^b^	2.20 ± 0.16^b^
	60 min	4.88 ± 0.29^a^	3.97 ± 0.66^ab^	3.09 ± 0.11 ^bc^	2.33 ± 0.16^c^	2.09 ± 0.31^c^
	24 hr	3.78 ± 0.70^a^	3.47 ± 0.36^ab^	3.16 ± 0.34 ^ab^	2.27 ± 0.13^ab^	2.03 ± 0.36^b^

Note

The different letters indicate significant differences in row (*p* <.05).

Regardless of thickness, the hydration ability of biofilms increased over time. The highest *SD* for NC and NW biofilms was recorded after 24 hr (Table 1). The greater surface area of nanoparticles compared with their relevant micro‐structures may vindicate their higher interaction with filler improving functional properties of the resultant material (Arora & Padua, 2010; De Azeredo, 2009; Lagaron et al., 2005; Radusin et al., 2016). The most rapid swelling rate was recorded for NW bio‐film (5.50 ± 1.04 g/g after 2 min). The high swelling capacity of NW biofilms makes it possible to incorporate water‐soluble active agents into their network during swelling. This characteristic is notable for the pad fabrication which is expected to be used in food active packaging.

### 3.2 WVP

**FIGURE 1:**
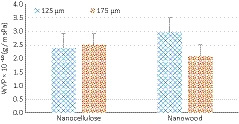
Water Vapor Permeability (WVP) of nanocellulose (NC) and nanowood (NW) biofilms at different thicknesses

Considering the central role of water in food deterioration, the WVP is one of the most important properties of biofilms. As shown in Figure 1, the film thickness affected the WVP properties. However, a significant effect was only found in the NW group (*p* <.05) where the highest WVP value was found at lower thicknesses (2.96 × 10^–10^ g/msPa at the thickness of 125 µm) facilitating their application for development of new pads.

### 3.3 Mechanical properties

Mechanical properties of biofilms (EB and TS) at different thicknesses are presented in Table 2. The results indicated that the thickness of biofilms could not exert considerable influences on the EB. The highest EB values of NC and NW biofilms were, however, found at the thickness of 175 µm (23.15% and 19.57%, respectively). The TS values of NC and NW were 1.32 and 0.70 MPa, respectively.

**TABLE 2:** Elongation at break (EB) and tensile strength (TS) properties of Nanocellulose (NC) and Nanowood (NW) biofilms

Biofilms	Thickness (µm)	Mechanical properties	
		Elongation at break (%)	Tensile strength (MPa)
Nanocellulose	125	18.53 ± 3.96^a^	1.16 ± 0.15^ab^
	175	23.15 ± 9.05^a^	1.32 ± 0.14^a^
	225	17.99 ± 4.73^a^	1.11 ± 0.08^ab^
	275	16.30 ± 2.33^a^	0.84 ± 0.09^b^
Nanowood	125	15.15 ± 2.27^a^	0.66 ± 0.02^a^
	175	19.57 ± 4.15^a^	0.70 ± 0.12^a^
	225	16.35 ± 6.30^a^	0.59 ± 0.12^a^
	275	13.75 ± 5.38^a^	0.45 ± 0.13^a^

Note

Values are mean ± SE. Different letters indicate significant differences (*p* <.05) among different thicknesses for each biofilm.

The EB and TS seem to have a nonlinear relationship with the thickness of the samples. The greatly improved EB of nano biofilms can be mainly due to the interfacial hydrogen and ion interaction between the polymer matrix and cellulose (Cao et al. 2008). Nevertheless, the mechanical performance of a specific composite is related to fiber traits, length, and production conditions (Cordeiro et al., 2011).

The mechanical properties of nano‐biopolymers in this study were comparable to the previously reported results for different types of cellulose nanofibers (Abdollahi et al., 2013; Abdollahi et al., 2013; Agustin et al., 2013; Azeredo et al., 2010; Chaichi et al., 2017; Fairley et al., 1996; Wan et al., 2009). In the investigation by Ljungberg et al. (2005), nanocomposite materials showed higher EB % and lower fragility compared to more aggregated samples, which is in accordance with our results.

### 3.4 XRD analysis

**FIGURE 2:**
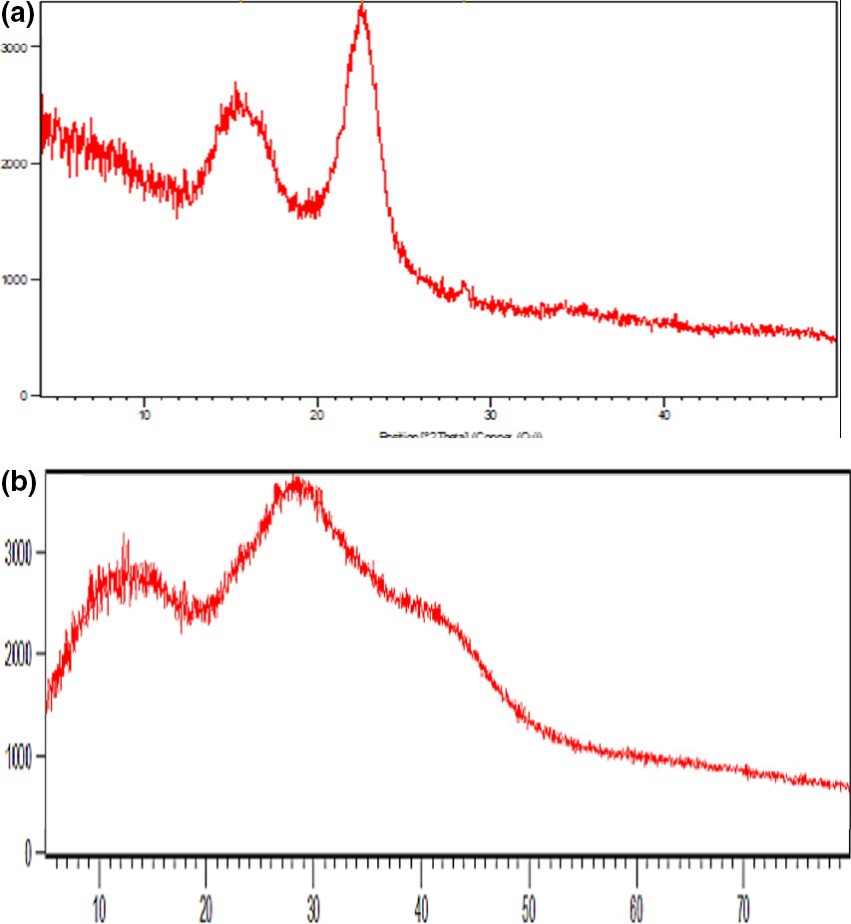
XRD patterns of (a) nanocellulose (NC); (b) nanowood (NW) biofilms

The XRD patterns of the NC and NW biofilms are shown in Figure 2. While NC showed two sharp peaks at 2θ = 15.6*º* and 22.51*º*, there was no sharp peak in diffractogram of NW. Three broad halo peaks at 2θ = 13.59*º*, 28.06*º*,and 42.90*º* observed in NW indicate an amorphous structure of NW which can, in turn, increase the hydration ability. These results are in accordance with the results of *SD* evaluation where NW samples had higher levels of *SD* compared with NC biofilms. The Piassava leaf has also been reported to comprise three broad halo peaks (Cordeiro et al., 2011). In the study conducted by Bodin et al. (2007), the diffractograms of NC biofilms were a combination of crystalline and amorphous peaks indicating their semi‐crystalline structures.

### 3.5 SEM analysis

**FIGURE 3:**
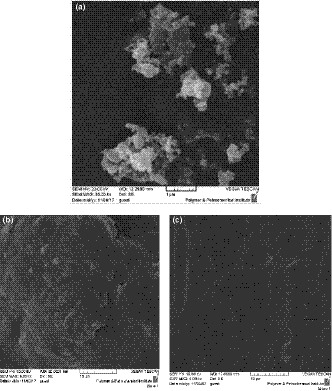
*SEM* micrographs of (a) nanowood (NW) particles; (b): surface of NW biofilm (thicknesses 125 µm); (c): cross‐section of NW biofilm (thicknesses 125 µm)

SEM allows an area of interest to be examined at different magnifications (1000–15000 x). The SEM image revealed the morphological changes in NW biofilms at different thicknesses. The particle size of NW ranged from 51.94 to 75.27 nm (Figure 3). The NW particles were highly aggregated, with some particles overlapping each other. The feature might be due to the strong hydrogen bonding of the particles and to the preparation of test specimens during the drying step (Habibi et al., 2010). Lu and Hsieh (2010) mentioned that the strong H‐bonding among cellulose nanocrystals (CNC) overcomes the repulsion of surface negative charges when CNC is in the dry phase. Scanning electron micrographs of the films showed homogeneous dispersion of NC in the starch matrix without any porosity. The NW biofilms had a denser matrix with good structural integrity at higher thicknesses.

Although the surface morphology of NW biofilms was different from their cross‐section structure at lower thicknesses, no differences were found at higher thicknesses.

### 3.6 EDXS analysis

EDXS can be applied in conjunction with *SEM* to explore the elemental composition of biofilms. EDXS spectra of NW biofilms were recorded in the binding energy region of 0–10 kV. Chemical elements determined in NW included C, O, and Si with an atomic percentage of 57.43%, 42.09%, and 0.48%, respectively (Table 3). The elemental composition of NW biofilms was, however, different from that of NW.

**TABLE 3:** Estimation of relative abundance of chemical elements in nanowood (NW) particles and biofilms at different thicknesses

Elements	Nanowood particles		Nanowood biofilms							
			Thickness (125 µm)				Thickness (175 µm)			
			Surface		Cross‐section		Surface		Cross‐section	
	Weight %	Atomic %	Weight %	Atomic %	Weight %	Atomic %	Weight %	Atomic %	Weight %	Atomic %
C	50.10	57.43	45.21	53.05	50.60	59.89	45.50	53.48	45.33	53.57
O	48.92	42.09	51.35	45.23	39.50	35.10	50.36	44.44	49.24	43.68
Si	0.98	0.48	3.43	1.72	9.90	5.01	4.15	2.08	5.44	2.75

At lower thicknesses, the surface and cross‐section of the films showed different element distribution. The higher O content of the surface of low‐thickness NW films can increase the moisture absorption vindicating the higher *SD*, WVP, and amorphous degree of these biofilms.

## 4 CONCLUSIONS

Two cellulose fibrils (NC and NW) were used to produce biofilms with various thicknesses. The properties of biofilms were affected by film thickness. Considering the high hydration ability and WVP of low‐thickness NW films, they are promising candidates to develop biodegradable films with the potential to be used as moisture absorbent pads in active food packaging. Furthermore, NW is directly produced from natural wood without using any chemical substances; the process is, therefore, environmentally friendly and green.

## CONFLICT OF INTREST

The authors declare that they have no conflict of interest.

## Data Availability

Data available on request from the authors.
